# A Rare Association of Bladder Endometriosis, Unicornuate Uterus, and Hematometra: A Case Report

**DOI:** 10.7759/cureus.117835

**Published:** 2026-10-13

**Authors:** Pranesh K Kumar, Vamshi C Yadav, Davneet Singh, Jagadeeshwar Thirukovela, Panduranga Rao Karnam

**Affiliations:** 1 Urology, Employees' State Insurance Corporation (ESIC) Medical College, Hyderabad, Hyderabad, IND

**Keywords:** bladder endometriosis, cyclical hematuria, deep pelvic endometriosis, hematometra, müllerian anomaly, unicornuate uterus

## Abstract

Endometriosis involving the urinary tract is uncommon, with the urinary bladder being the most frequently affected site. Its nonspecific urinary manifestations may result in delayed diagnosis. Congenital Müllerian anomalies, particularly a unicornuate uterus with a noncommunicating functional rudimentary horn, may be associated with hematometra and endometriosis secondary to obstructed menstrual outflow and retrograde menstruation. We report an unusual coexistence of bladder endometriosis, a right unicornuate uterus with a noncommunicating left rudimentary cavity, and hematometra in a 22-year-old nulliparous woman who presented with a one-year history of cyclical dysuria, hematuria, and dyspareunia. She had regular menstrual cycles associated with dysmenorrhea and had attempted conception without success. Pelvic magnetic resonance imaging demonstrated a right hemi-uterus measuring approximately 7 × 3 × 2.5 cm and a separate left-sided endometrial cavity measuring 4.1 × 3.5 × 4.2 cm, which did not communicate with the uterus and was distended with hematometra. An ill-defined T2 hypointense lesion measuring 3.4 × 2.3 × 3 cm with T1 hyperintense foci was centered in the vesicouterine pouch, extending posteriorly into the lower uterine segment and cervix and anteriorly into the bladder wall with intraluminal extension. Thickening of the proximal bilateral round ligaments was also noted, with the overall appearance suggestive of deep pelvic endometriosis. Cystoscopy showed multiple reddish-brown cystic lesions involving the lateral and posterior walls of the urinary bladder. Near-complete transurethral resection of the bladder lesions was performed, along with laparoscopic drainage and partial excision of the rudimentary horn. Histopathological examination of the resected bladder lesions confirmed bladder endometriosis. Postoperatively, the patient was started on leuprolide acetate and remained symptom-free at six-month follow-up. This case highlights the importance of considering bladder endometriosis in young women presenting with cyclical urinary symptoms and demonstrates the value of magnetic resonance imaging in delineating Müllerian anomalies, hematometra, and the extent of deep pelvic endometriosis. The coexistence of extensive bladder endometriosis with a unicornuate uterus and hematometra underscores the importance of coordinated urological and gynecological management.

## Introduction

Endometriosis is a chronic inflammatory condition characterized by the presence of endometrial-like tissue outside the uterine cavity. Although it predominantly affects the pelvic reproductive organs, extragenital involvement can occur. Urinary tract endometriosis is uncommon, occurring in approximately 1% of patients with endometriosis, with the urinary bladder being the most frequently affected site [1,2]. Bladder endometriosis may present with dysuria, urinary frequency, pelvic pain, and hematuria. Although cyclical hematuria is considered a characteristic manifestation, it occurs in only a minority of patients. Consequently, urinary tract endometriosis may be overlooked or misdiagnosed as a more common urological condition [1,2].

Bladder endometriosis is frequently associated with deep infiltrating endometriosis involving the anterior pelvic compartment. Magnetic resonance imaging (MRI) is valuable for detecting and characterizing deep infiltrating endometriosis and for determining its relationship with adjacent pelvic organs. MRI can demonstrate the location, depth, and extent of bladder involvement and therefore plays an important role in preoperative planning [3].

Congenital Müllerian anomalies represent a spectrum of developmental abnormalities of the female reproductive tract. A unicornuate uterus results from incomplete development of one Müllerian duct and may be associated with a rudimentary horn. When a functional rudimentary horn does not communicate with the main uterine cavity, menstrual blood may accumulate within it, resulting in hematometra. Obstructed menstrual outflow and retrograde menstruation may contribute to the development of endometriosis and may also have implications for fertility [4,5].

The coexistence of a unicornuate uterus with a noncommunicating rudimentary horn, hematometra, and bladder endometriosis is uncommon, with only a limited number of cases described in the literature. A previous report documented a unicornuate uterus with a noncommunicating rudimentary horn containing hematometra and a communicating bladder endometriotic nodule [6]. Extensive bladder involvement in this setting may pose diagnostic and therapeutic challenges because of the proximity of the bladder to the uterus and other pelvic structures. Recognizing the underlying Müllerian anomaly is therefore important for defining the extent of disease and planning appropriate treatment. The present case describes extensive deep pelvic endometriosis involving the urinary bladder, with intraluminal extension, in association with a unicornuate uterus and hematometra. We discuss the clinical, radiological, cystoscopic, operative, and histopathological findings, highlighting the diagnostic and therapeutic considerations in this unusual presentation.

## Case presentation

A 22-year-old nulliparous woman presented with a one-year history of dysuria, hematuria, and dyspareunia. Her urinary symptoms were present throughout the menstrual cycle but worsened during menstruation. She reported regular menstrual cycles associated with dysmenorrhea and had attempted conception but had not conceived. The cyclical exacerbation of her urinary and pelvic symptoms raised clinical suspicion of endometriosis.

On abdominal examination, the patient had mild pelvic tenderness, with no other significant findings. Initial investigations included an unremarkable urinalysis and a urine culture showing no bacterial growth. Ultrasonography of the abdomen revealed multiple small bladder masses, a unicornuate uterus, and hematometra, prompting further evaluation with magnetic resonance imaging (MRI).

MRI of the pelvis with plain and contrast sequences demonstrated a right-sided hemi-uterus measuring approximately 7 × 3 × 2.5 cm, consistent with a unicornuate uterus. A separate left-sided endometrial cavity measuring approximately 4.1 × 3.5 × 4.2 cm did not communicate with the main uterine cavity and was distended with hematometra, as seen in Figure 1.

**Figure 1:**
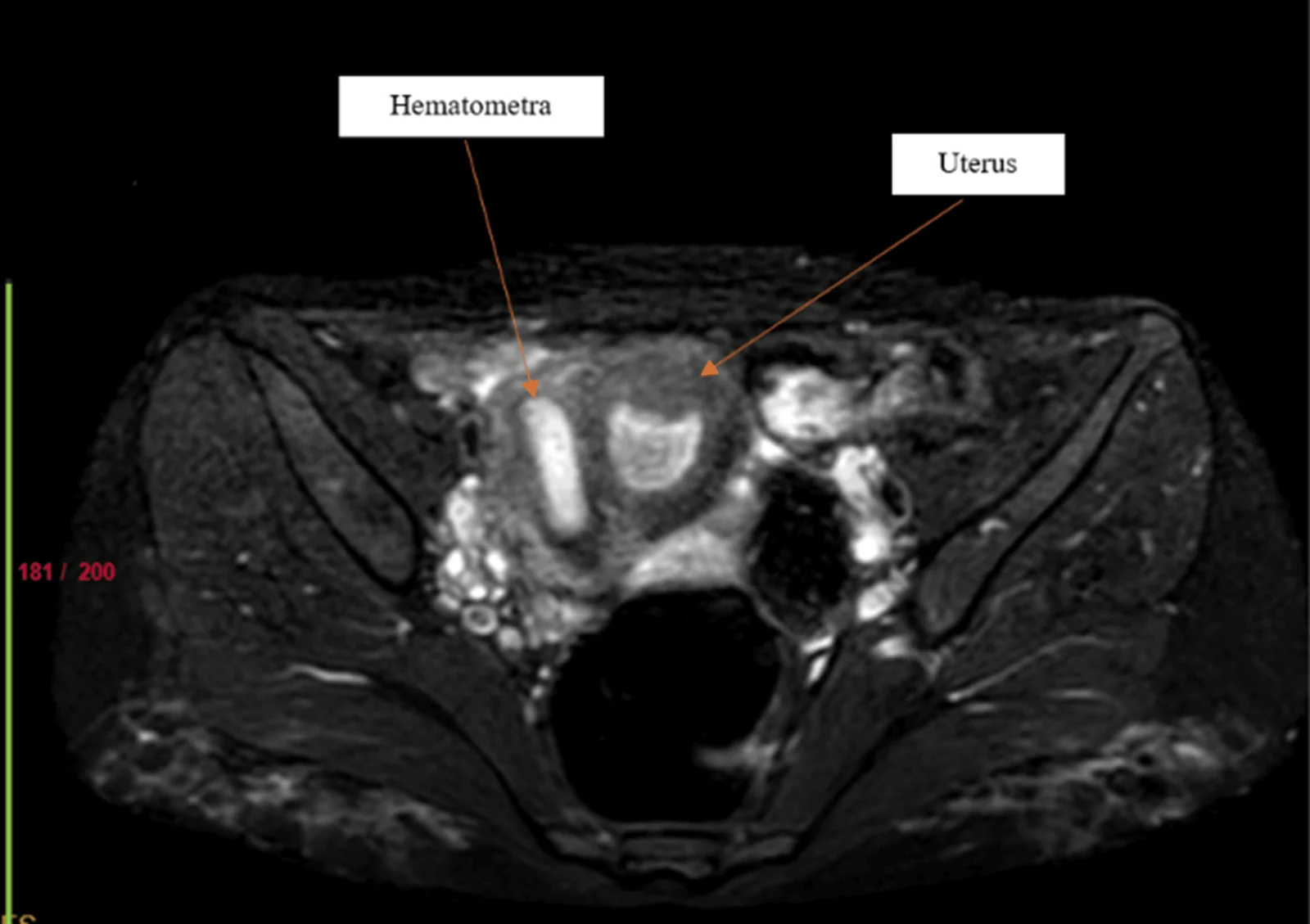
Pelvic MRI demonstrating a unicornuate uterus and a noncommunicating rudimentary uterine cavity containing hematometra.

MRI also demonstrated an ill-defined T2-hypointense lesion measuring approximately 3.4 × 2.3 × 3 cm, containing T1-hyperintense foci and centered in the vesicouterine pouch. The lesion extended posteriorly into the lower uterine segment and cervix, and anteriorly into the bladder wall and lumen. It demonstrated mild heterogeneous enhancement following contrast administration (Figure 2). Focal thickening of the junctional zone along the right lateral uterine wall, measuring approximately 1.5 cm, was considered possibly consistent with focal adenomyosis. Thickening of the proximal portions of both round ligaments was also noted.

**Figure 2:**
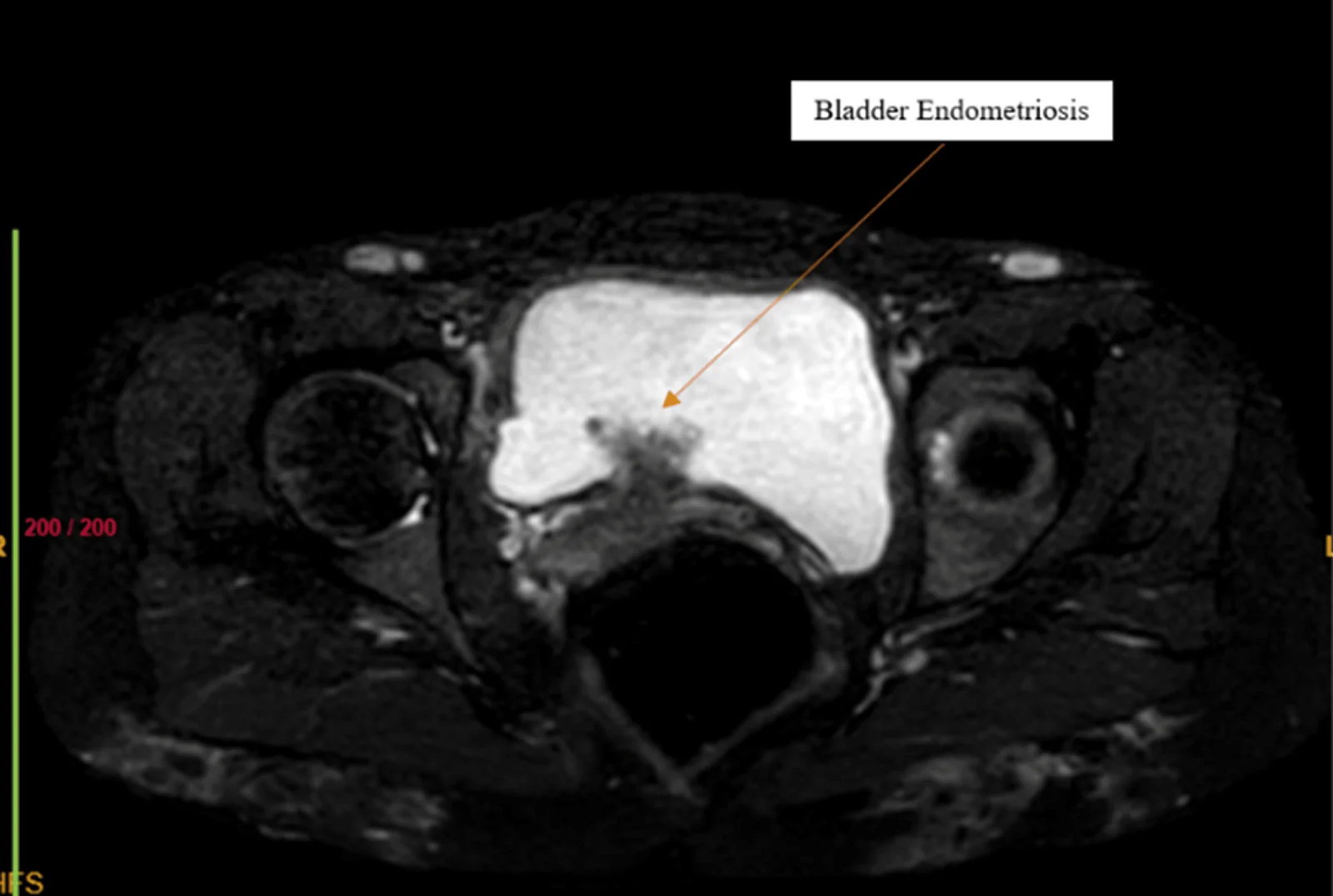
Axial pelvic MRI demonstrating an infiltrative lesion centered in the vesicouterine pouch, extending into the bladder wall and lumen, with posterior extension towards the lower uterine segment and cervix.

The right ovary measured approximately 3 × 1.8 cm and contained small subcentimetric T1-hyperintense cystic lesions. The left ovary measured approximately 4 × 1.7 cm and contained multiple cystic lesions, including a T1-hyperintense cyst. These findings were considered possibly representative of endometriotic cysts. No free fluid or pelvic or inguinal lymphadenopathy was identified.

Cystoscopy subsequently demonstrated multiple reddish-brown cystic lesions within the urinary bladder, predominantly involving the lateral and posterior walls (Figure 3).

**Figure 3:**
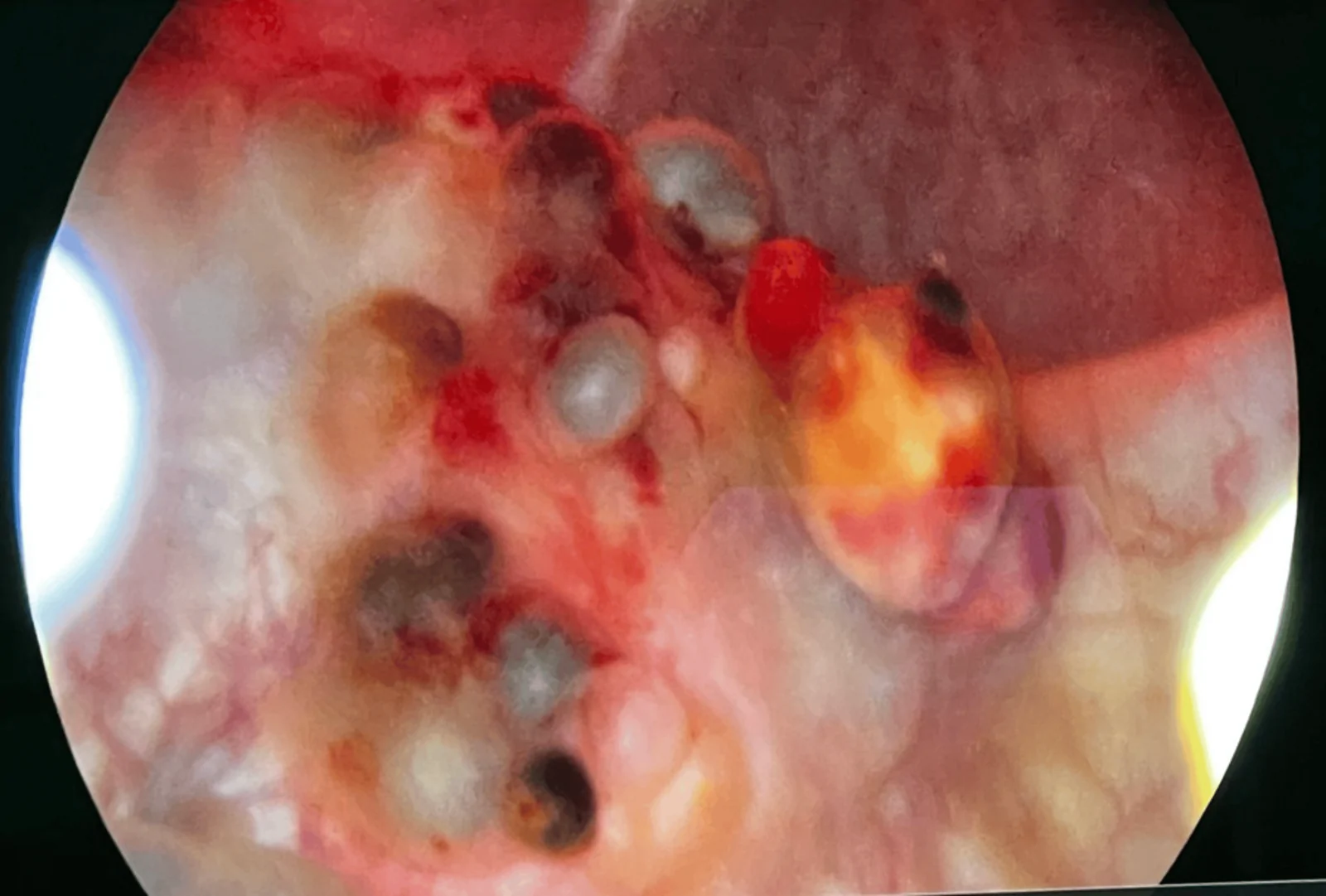
Cystoscopic appearance of multiple reddish-brown lesions involving the lateral and posterior walls of the urinary bladder.

In view of the imaging and cystoscopic findings, transurethral resection of the bladder lesions was performed, achieving near-complete resection (Figure 4).

**Figure 4:**
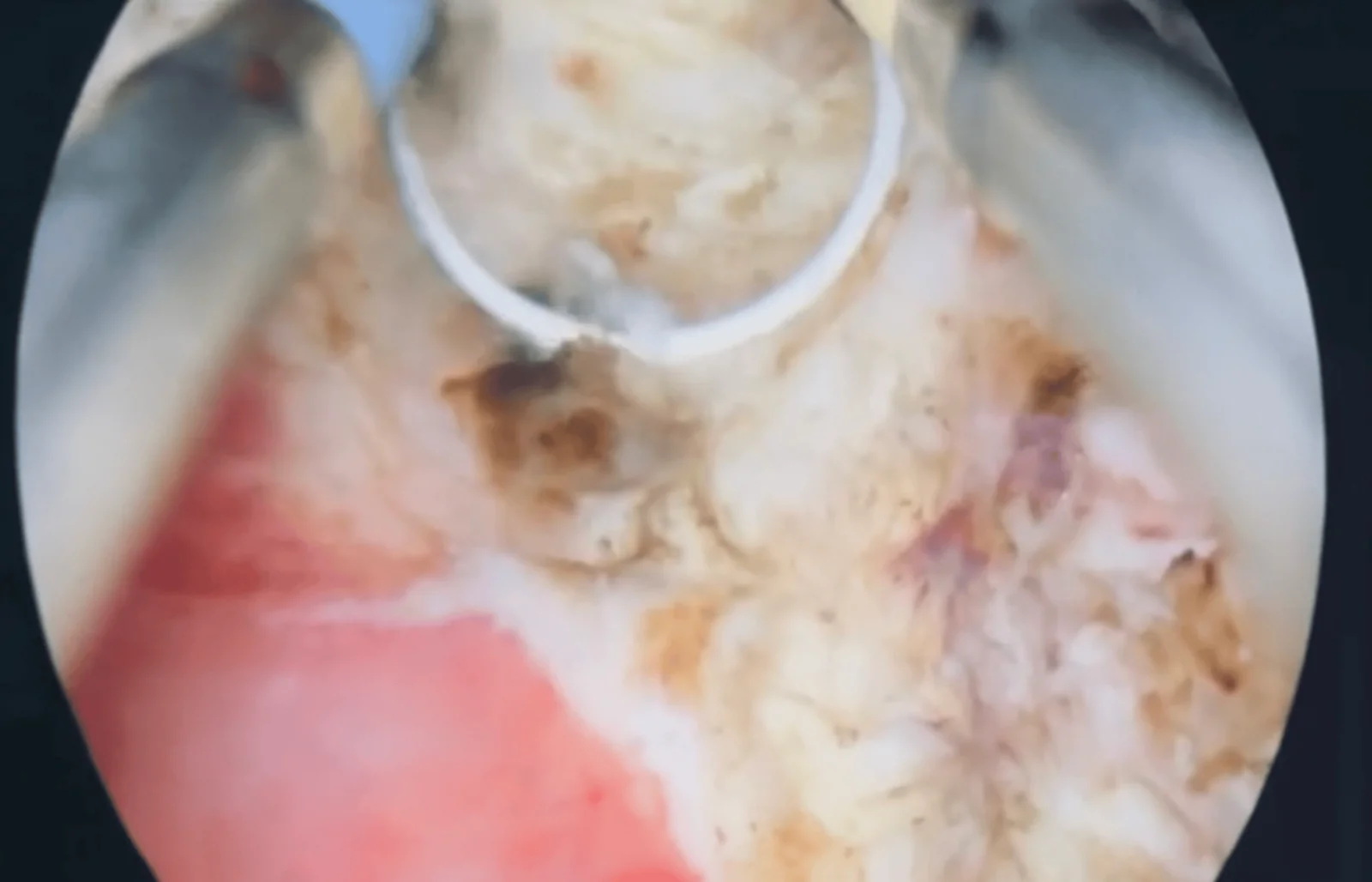
Intraoperative cystoscopic view during transurethral resection of the bladder lesions.

During the same treatment episode, the patient underwent laparoscopic drainage of the hematometra and partial excision of the rudimentary horn (Figure 5). Complete excision of the horn was not performed because of extensive adhesions and technical difficulty. The excised rudimentary horn was not submitted for histopathological examination.

**Figure 5:**
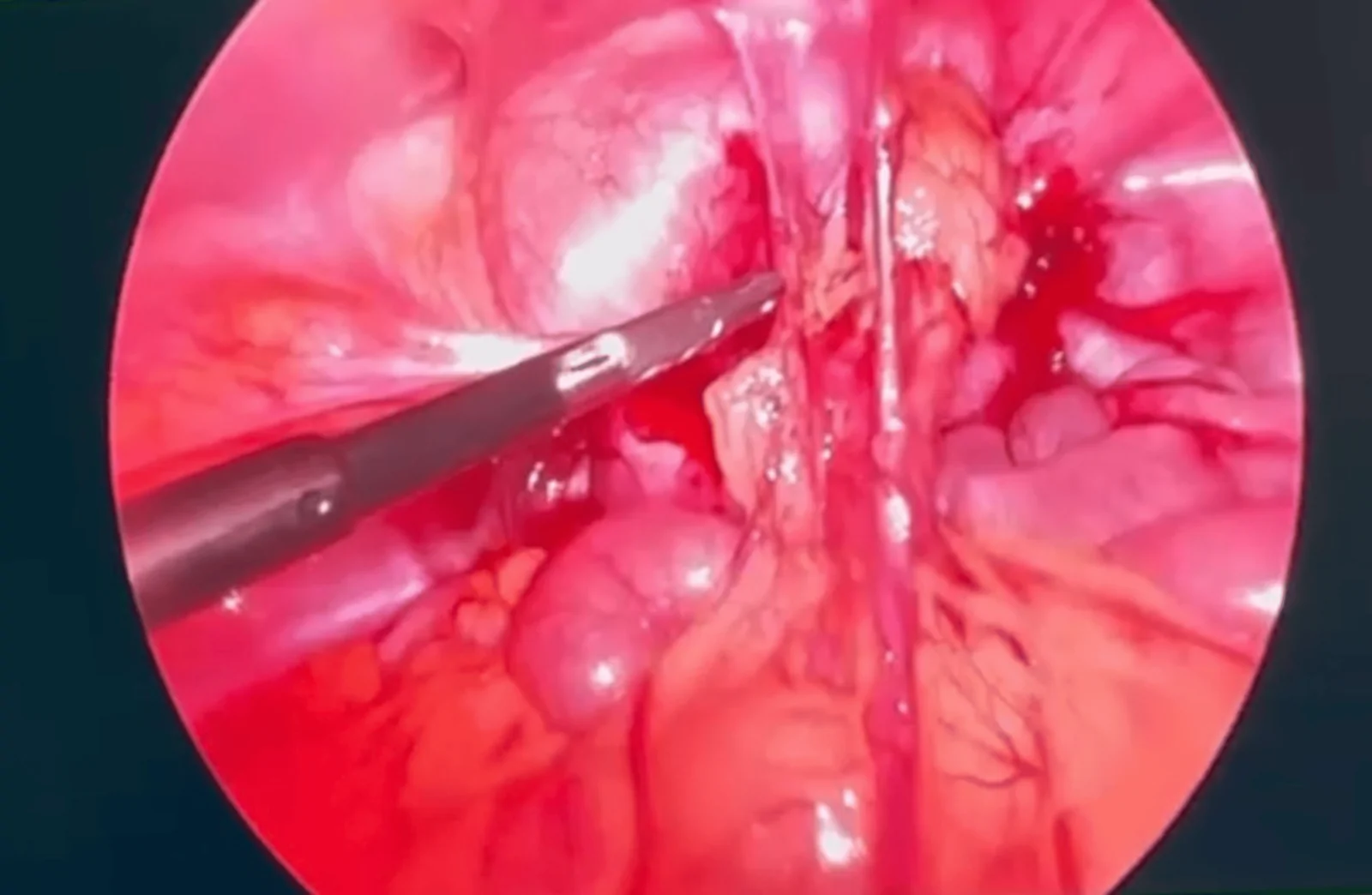
Intraoperative laparoscopic view following drainage of the hematometra and partial excision of the rudimentary uterine horn.

Histopathological examination of the resected bladder tissue confirmed bladder endometriosis (Figure 6). Postoperatively, the patient received leuprolide acetate 11.25 mg intramuscularly every three months for a total of six months.

**Figure 6:**
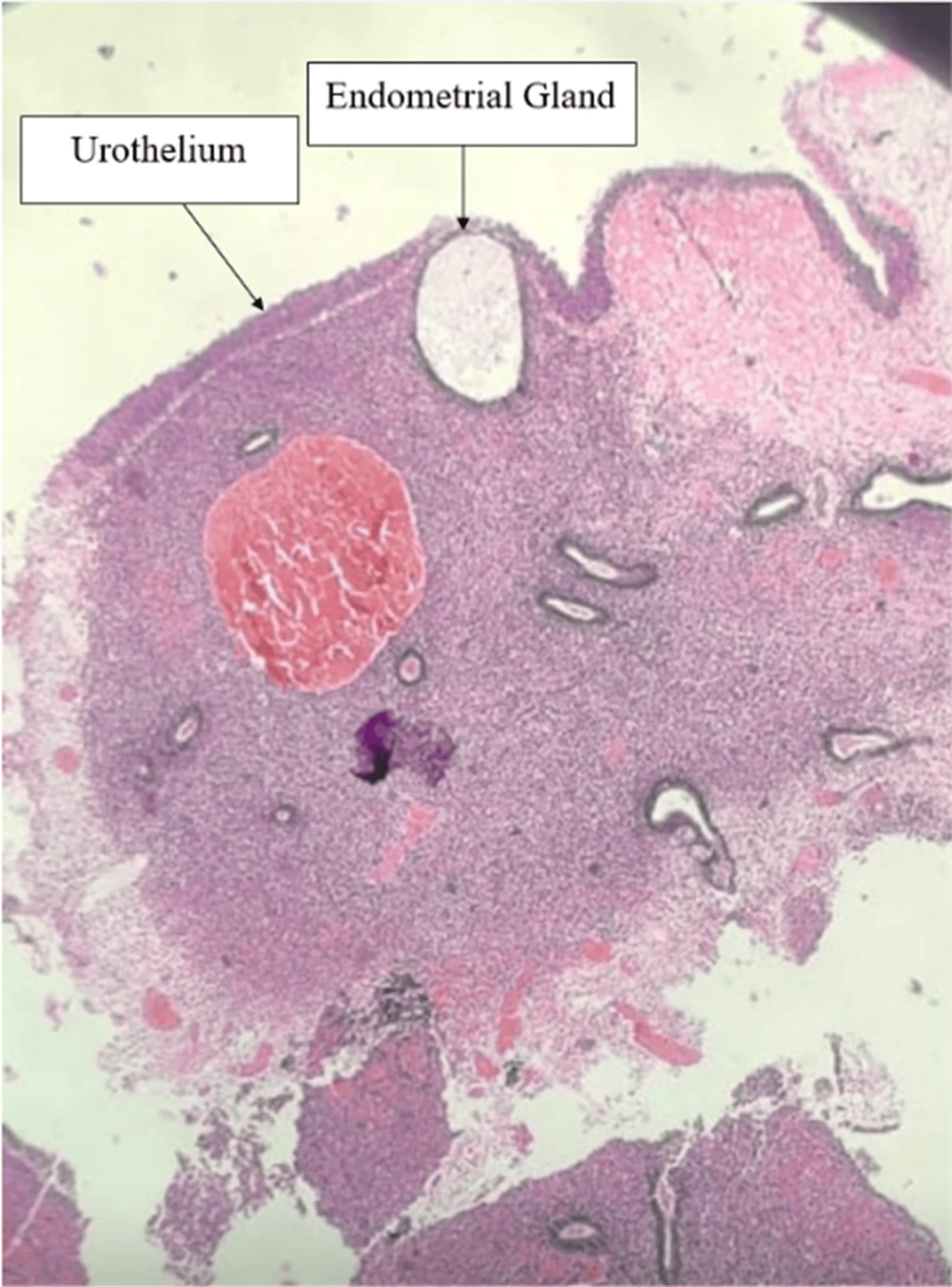
Histopathological examination of the resected bladder lesion demonstrating endometrial tissue, confirming bladder endometriosis.

At the six-month follow-up, the patient was asymptomatic. Repeat ultrasonography, cystoscopy, and urinalysis were normal, with no evidence of recurrent bladder lesions on cystoscopy. Follow-up MRI was not performed. The patient expressed a desire to attempt conception at a later date; therefore, no subsequent fertility outcome was available at the time of follow-up (Table 1).

**Table 1:** Clinical timeline of presentation, diagnostic evaluation, treatment, and follow-up.

Stage	Clinical event
Approximately one year before presentation	Onset of dysuria, hematuria, and dyspareunia, with worsening during menstruation
Initial evaluation	Urinalysis, urine culture, and abdominal ultrasonography
Further evaluation	Pelvic MRI demonstrating the uterine anomaly, hematometra, and infiltrative vesicouterine lesion
Diagnostic evaluation	Cystoscopy demonstrating multiple bladder lesions
Surgical management	Near-complete transurethral resection of the bladder lesions, laparoscopic drainage of hematometra, and partial excision of the rudimentary horn
Postoperative management	Leuprolide acetate initiated
Six-month follow-up	Patient remained symptom-free

## Discussion

Urinary tract endometriosis is an uncommon manifestation of endometriosis, with the urinary bladder being the most frequently affected site [1,2]. Clinical manifestations include dysuria, urinary frequency, urgency, suprapubic or pelvic pain, and hematuria. Symptoms may be cyclical or noncyclical, and the absence of a clear temporal relationship with menstruation can contribute to diagnostic delay [1,2]. Cyclical hematuria is a characteristic manifestation of bladder endometriosis but occurs in only a minority of affected patients. Recognizing a menstrual association with urinary symptoms is therefore clinically important. In the present case, dysuria and hematuria occurred throughout the menstrual cycle but worsened during menstruation, accompanied by dyspareunia and dysmenorrhea. The initial urinalysis was unremarkable, and urine culture showed no bacterial growth. These findings, together with the imaging abnormalities, supported further evaluation for a noninfectious cause of the urinary symptoms.

Bladder endometriosis commonly involves the posterior bladder wall and base and may extend into the detrusor muscle. Lesions may appear as nodular or infiltrative abnormalities containing hemorrhagic components. Magnetic resonance imaging (MRI) is particularly useful for characterizing these lesions, defining their relationship with adjacent pelvic structures, and planning treatment [3]. In this patient, MRI demonstrated an ill-defined T2-hypointense lesion containing T1-hyperintense foci, centered in the vesicouterine pouch and extending posteriorly towards the lower uterine segment and cervix and anteriorly into the bladder wall and lumen. Thickening of the proximal portions of both round ligaments further supported the radiological impression of deep pelvic endometriosis. Ultrasonography had initially identified multiple small bladder masses and the associated uterine anomaly, while MRI provided more detailed anatomical characterization of the pelvic disease.

The MRI also demonstrated a right-sided hemi-uterus and a separate noncommunicating left-sided cavity containing hematometra. A unicornuate uterus results from incomplete development of one Müllerian duct and may be associated with a rudimentary horn. The European Society of Human Reproduction and Embryology (ESHRE)/European Society for Gynaecological Endoscopy (ESGE) classification categorizes a unicornuate uterus as a hemi-uterus [4]. When a functional rudimentary horn does not communicate with the main uterine cavity, menstrual blood may accumulate within it, resulting in hematometra. This may cause dysmenorrhea and chronic pelvic pain and may be associated with endometriosis and reproductive difficulties [5]. In the present case, the noncommunicating cavity containing hematometra was identified on MRI and subsequently treated surgically.

The coexistence of a noncommunicating rudimentary horn, hematometra, and endometriosis raises the possibility of an association between obstructed menstrual outflow and the development of endometriosis. Retrograde menstruation is one proposed mechanism by which menstrual material may reach the peritoneal cavity and contribute to endometriotic implantation [5]. However, the mechanism underlying the coexistence of these abnormalities cannot be established from an individual case, and the presence of hematometra does not by itself demonstrate that it caused the bladder endometriosis.

A particularly relevant report by Giampaolino et al. described a patient with a unicornuate uterus, a noncommunicating rudimentary horn containing hematometra, and a communicating bladder endometriotic nodule [6]. That patient presented with dysmenorrhea, cyclical dysuria, hematuria, and infertility. The report illustrates the potential coexistence of obstructive Müllerian anatomy and bladder endometriosis. In the present case, MRI demonstrated a relatively extensive infiltrative lesion centered in the vesicouterine pouch, with extension into the lower uterine segment and cervix and through the bladder wall into the bladder lumen. Cystoscopy revealed multiple reddish-brown cystic lesions predominantly involving the lateral and posterior bladder walls. Histopathological examination of the resected bladder tissue confirmed endometriosis. The multifocal bladder involvement and the need for near-complete transurethral resection distinguish the clinical presentation from the previously reported case involving a solitary communicating bladder nodule.

Cystoscopy provides direct visualization of bladder lesions and permits tissue sampling for histopathological diagnosis. Endometriotic lesions may appear bluish, reddish, or brown because of associated hemorrhagic components [2,3]. In this patient, cystoscopy demonstrated multiple reddish-brown cystic lesions, and histopathological examination confirmed bladder endometriosis. Near-complete transurethral resection was performed, with no visible residual lesions documented at the end of the procedure. The agreement between the clinical presentation, MRI findings, cystoscopic appearance, and histopathological diagnosis supported the final diagnosis.

Treatment of bladder endometriosis should be individualized according to symptom severity, lesion location and depth, associated pelvic disease, and reproductive goals. Hormonal treatment may suppress endometriotic activity and improve symptoms, whereas surgery may be indicated when symptoms are significant, lesions require histopathological diagnosis, or anatomical involvement warrants intervention [1,2,7]. Surgical approaches include transurethral resection in selected cases and more extensive excisional procedures, such as partial cystectomy, depending on the location and depth of disease [2,7]. Management of a symptomatic, functional, noncommunicating rudimentary horn generally involves surgical treatment, particularly when hematometra or associated endometriosis is present [5].

In the present case, near-complete transurethral resection of the bladder lesions was combined with laparoscopic drainage of the hematometra and partial excision of the rudimentary horn. Complete excision of the horn was not feasible because of adhesions and technical difficulty. The horn was not submitted for histopathological examination; therefore, no histological conclusions can be drawn regarding that structure. Postoperatively, the patient received leuprolide acetate at a dose of 11.25 mg every three months for six months. At the six-month follow-up, she was asymptomatic, and ultrasonography, cystoscopy, and urinalysis were normal. These findings indicate a favorable short-term clinical outcome. However, no follow-up MRI was performed, and the absence of symptoms and normal cystoscopy do not establish complete resolution of extravesical endometriotic disease. The patient planned to attempt conception at a later date, so a subsequent fertility outcome was not available.

This report has several limitations. As a single case, it cannot establish a causal relationship between the Müllerian anomaly, hematometra, and bladder endometriosis. The rudimentary horn was not submitted for histopathological examination, and follow-up MRI was not performed. Although the patient remained asymptomatic with normal ultrasonography, cystoscopy, and urinalysis at six months, the limited follow-up period prevents conclusions regarding long-term recurrence, persistence of extravesical disease, or subsequent fertility outcomes.

This case highlights the importance of considering bladder endometriosis in young women presenting with urinary symptoms that worsen during menstruation, particularly when initial urine investigations do not support infection. The presence of a Müllerian anomaly with hematometra should prompt careful assessment for associated pelvic endometriosis. Ultrasonography may identify the initial anatomical abnormalities, while MRI provides more detailed mapping of the uterine anomaly and the extent of pelvic disease. Cystoscopy and histopathological examination can establish the diagnosis of bladder involvement. Coordinated urological and gynecological management can address both urinary tract disease and the associated Müllerian anomaly while taking into account the patient's reproductive plans.

## Conclusions

Bladder endometriosis should be considered in young women presenting with cyclical urinary symptoms, particularly when associated with dysmenorrhea and dyspareunia. A unicornuate uterus with a functional noncommunicating rudimentary horn may be complicated by hematometra and associated endometriosis. MRI is valuable for delineating the Müllerian anomaly, characterizing hematometra, and mapping the extent of deep pelvic and bladder endometriosis. Cystoscopy and histopathological examination provide important confirmation of bladder involvement. A coordinated urological and gynecological approach combining surgical treatment with postoperative hormonal suppression can achieve effective symptom control in selected patients.
